# Genetic Assessment and Positioning of Algerian Barley Landraces with Respect to Landraces from the Middle East and Europe Using RAPD and SSR Markers

**DOI:** 10.3390/cimb46120852

**Published:** 2024-12-17

**Authors:** Hamama Guetteche, Ali Jarrar, Sascha Wetters, Leila Rouabah, Abdelkader Rouabah, Abdelkader Benbelkacem, Ruzanna Sadoyan, Adnan Kanbar, Peter Nick

**Affiliations:** 1 Molecular and Cellular Biology Laboratory, University of Mentouri Constantine I, Constantine 2500, Algeria; guetteche_hamama@hotmail.fr (H.G.); leilarouabah27@yahoo.fr (L.R.); abdourouabah@hotmail.fr (A.R.); 2 Molecular Cell Biology, Joseph Gottlieb Kölreuter Institute for Plant Sciences, Karlsruhe Institute of Technology, 76131 Karlsruhe, Germany; alijarrar8080@gmail.com (A.J.); sascha.wetters@kit.edu (S.W.); kanbaradnan@gmail.com (A.K.); 3 National Institute of Agronomic Research of Algeria, Algiers 16200, Algeria; abdelkader.benbelkacem@inraa.dz; 4 Laboratory of Biologically Active Compounds and Biosafety, Armenian State Pedagogical University Named After Kh, Yerevan 0010, Armenia; ruzannasad@mail.ru

**Keywords:** barley, landraces, genetic diversity, molecular markers, RAPD, SSR, multi-omics, molecular phylogeny, molecular breeding

## Abstract

Landraces are a critical genetic resource for resilience breeding, offering solutions to prepare agriculture for the challenges posed by climate change. Their efficient utilisation depends on understanding their history and genetic relationships. The current study investigates the phylogenetic relationships of barley landraces from Algeria, varieties from the Near and Middle East, traditional landraces, and modern cultivars from Europe. Using a core set of 33 varieties, including the wild ancestor *Hordeum spontaneum* from Armenia, genetic diversity was analysed with Random Amplified Polymorphic DNA (RAPD) and Simple Sequence Repeat (SSR) markers spanning all barley chromosomes. Based on the SSR-based phylogeny, the Algerian varieties are well clustered with those from the Near East, while distinct from the European varieties. The findings from RAPD markers partially support these results. Using exclusively traditional landraces, where a region of origin can be defined, the SSR markers are analysed separately for each chromosome individually, and the resulting clades are represented by the respective region of origin. This strategy resolves qualitative differences in geographic resolution, depending on the chromosome. While marker HvB23D (chromosome 4) separated the wild *H. spontaneum* from all domesticated genotypes, markers Bmag19 and Hv13GIII (chromosome 3) reveal four distinct geographic clusters (Maghreb, Near and Middle East, West Europe, Central Europe). These biogeographic patterns suggest a model, where divergence of domesticated barley due to human activity interacted with introgression of individual chromosomes from wild barley, yielding adaptive diversity. These biogeographic patterns suggest a model in which the divergence of domesticated barley, driven by human activity, interacts with the introgression of chromosomes from wild barley, resulting in the creation of adaptive genetic diversity. Our research advances our knowledge of barley landraces’ functional genomics and highlights their potential in molecular breeding, particularly for developing resilient varieties suited to diverse environmental conditions.

## 1. Introduction

Barley (*Hordeum vulgare* ssp. *vulgare* L.) is the crop with the longest documented history of domestication. Archaeological studies estimate that barley cultivation began more than 10,000 years ago in the Fertile Crescent [1]. Excavations in the South Anatolian temple of Göbekli Tepe show the use of large quantities of *H. vulgare* ssp. *spontaneum* (the wild ancestor of domesticated barley) for ritual use [2]. Barley was a staple food for our ancestors, playing a crucial role in the transition from nomadic hunting and gathering to settled farming during the Neolithic revolution. Through their skill in cultivating and domesticating barley and other cereals, early farmers became the first true breeders in this sense [3].

Barley has been an important crop, ranking fourth among all cultivated crops worldwide, with a total surface of nearly 50 million hectares, and a production of more than 145 million tons [4]. In addition to its use as food, barley plays an important role for animal feed, malting, and brewing. The impact of these uses may vary depending on the region. While malting and brewing are prevalent in Europe and East Asia, they have less economic weight in North Africa or the Near and Middle East [5]. Although the objectives for new varieties differ, due to these differences in usage, the pressure for higher yield resulting from the rapid population growth, and the need for resistant varieties which can be grown and adapt to climate change are common topics in barley breeding [6,7].

Barley comprises a substantial genetic diversity with nearly 400,000 accessions assembled in different worldwide germplasms [8]. This remarkable genetic diversity is the result of different evolutionary processes including natural and human selection, but also migrations, mutations, and introgression from the wild progenitors of barley into traditional landraces [9]. Unlike wheat, where the crop wild relatives differ in ploidy, both, the domesticated *H*. *vulgare* ssp. *vulgare*, and the wild ancestor *H. vulgare* ssp. *spontaneum* are diploids, which facilitates gene flow and introgression in both directions. Barley breeding made ample use of this gene flow to introduce traits of interest. A central role has been the resistance to Powdery Mildew (for instance through the monogenic *mlo* mutation), which motivated the extension of the genetic pool even further to neighbouring species, such as *H. bulbosum* [10]. As a result, barley has become a systematic experimental model, and such crosses have enhanced its diversity even further.

Nevertheless, the strife to develop elite crops and a selection bias upon high yield has drastically narrowed variability and eroded genetic diversity [11]. Prominently, potentially useful genes that are substantial and involved in the resilience of barley to abiotic and biotic stress have been lost, an urging demand to counter the consequence of climate change, have been neglected and often lost. These new cultivars are performing well under optimal conditions. However, they fail under stress [11]. In contrast, many of the old varieties and even more their wild ancestors, have retained such resilience and, therefore, are able to provide yield under stress. To neglect those ancient resources, will inevitably impact food security in the long term.

Marker-assisted breeding can help to re-integrate those neglected sources of genetic variation, as it supports detection of a locus of interest upon introgression into the background of a given elite variety [12]. Furthermore, it allows quantifying the diversity, plotting the origins, and computing the phylogenetic relations, necessary for understanding the evolution and following the history of a donor genotype. Since the importance of genetic diversity has become evident, the study and survey of barley diversity has attracted attention. This includes the need to evaluate the variation in the available germplasm and to save these resources for future application [13].

The current work aims to screen genetic variation in landraces of *H. vulgare* to understand the history of two landraces, Saïda183 and Tichedrett, relevant for Algeria. We compare the phylogenetic relationship between those landraces with traditional varieties from the Middle East and Central Europe, as well as with wild barley (*H. spontaneum*) using Random Amplified Polymorphic DNA (RAPD) and Simple Sequence Repeats (SSR) markers. We arrive at a model, where migration of domesticated barley, migration of wild barley (probably as weedy contaminant), gene flow between them, and local differentiation contributed to the specific genotypes of these North African landraces. This graphical representation will enhance our chromosome-level findings, highlighting the contrasting patterns of domestication and introgression while underscoring the role of specific alleles in regional adaptations. Ultimately, these insights will be relevant for future multi-omics studies aimed at harnessing the genetic diversity of barley for molecular breeding applications.

## 2. Materials and Methods

### 2.1. Plant Material

The study included a total number of 33 barley accessions including one accession of wild barley (*H. spontaneum*) from Armenia, 12 presumably autochthonous landraces, a couple of traditional registered varieties, and modern cultivars originating from complex crosses. For the details on these accessions, their source, pedigree, and source references, as far as available (Table 1).

### 2.2. Extraction of DNA

Leaves from all 33 accessions were harvested after the fifth leaf stage, immediately frozen in liquid nitrogen, and subsequently homogenised using a TissueLyser system (Qiagen subsequently, Hilden, Germany). DNA was extracted from 60 mg aliquots of the homogenised material using the Invisorb^®^ Spin Plant Mini Kit (Stratec Biomedical AG, Birkenfeld, Germany). The purity and concentration of the extracted DNA were assessed using a NanoDrop spectrophotometer (Peqlab, Erlangen, Germany). DNA concentrations were then adjusted to 50–80 µg/µL and stored at −20 °C until further analysis.

### 2.3. PCR Amplification and Electrophoresis Gel

To screen the genetic diversity of the accessions (Table 1), we used a set of ten Randomly Amplified Polymorphic DNA (RAPD) markers (Table 2) and ten Simple Sequence Repeat (SSR) markers (Table 3) for PCR from a template of 1 µg of genomic DNA. The primer sequences for the markers were chosen based on their use in studies cited in the references provided in Table 2 and Table 3. Reaction volumes of 20 µL were prepared with nuclease-free water Biozym, Lonza containing 2 µL 1 × Thermopol Buffer (New England Biolabs, Ipswich, MA, USA), 1 µL of bovine serum albumin (1 g·L^−1^), 200 mM dNTPs (New England Biolabs), 0.2 units of Taq polymerase (New England Biolabs) and either 0.4 mM of each RAPD primers or 0.2 mM for each the forward and reverse primer in the case of SSR.

To amplify the RAPDs, we used 45 cycles of initial denaturation at 94 °C for 1 min, annealing at 36 °C for 1 min, and extension at 68 °C for 2 min, adding a final extension step of 5 min at 68 °C. We amplified the SSR markers by initial denaturation at 95 °C for 5 min, followed by 30 cycles at 94 °C for 1 min, annealing for 1 min between 52 °C and 60 °C, depending on the different primer combinations for 1 min, extension at 68 °C for 1 min, ending with an extension of 68 °C for 5 min. Each PCR amplification was repeated three time to validate the patterns.

We separated the RAPD products by electrophoresis in a 1% agarose gel with 5% *v*/*v* SYBR Safe (Invitrogen, Thermo Fisher Scientific, Dreieich, Germany) at a voltage of 100 V, visualising by excitation with blue light against a 100 bp DNA ladder (NEB) as size marker. To improve the resolution for the SSR amplicons, we used a higher concentration of agarose (2% *w*/*v*) and a longer running time (45 min) at 130 V.

### 2.4. Data Analysis

The banding pattern was converted into a binary matrix by assigning a score of 1 if a band was present at a given size and 0 if it was absent. The decision to use a 0/1 matrix for SSR markers was intentional to standardise the data format for subsequent analyses, including genetic diversity assessment, cluster analysis, and testing a novel approach that combines both results. This binary matrix was then used to calculate a pairwise similarity matrix, which enabled the computation of Jaccard indices using Past 3.22 software [29].

The polymorphism information content (PIC) was calculated for the dominant markers RAPD each marker according to [30], as follows:

PIC = 2 fi (1 − fi)

where fi is the frequency of the amplified allele (band present), and 1 − fi is the frequency of the null allele.

For SSR markers the PIC value was calculated with the equation proposed by Botstein et al. [31]:

$$PIC=1-\sum_{j=1}^{n}{P_{ij}}^{2}$$

where *P_ij_* is the frequency of a particular band *j*, obtained from either the SSR or the RAPD patterns for marker *i*, and *n* the entire sum of bands; *P_i_* and *P_j_* are the population frequency of the ith and *j*th alleles and *n* = number of alleles, the entire sum of bands.

**Figure 1:**
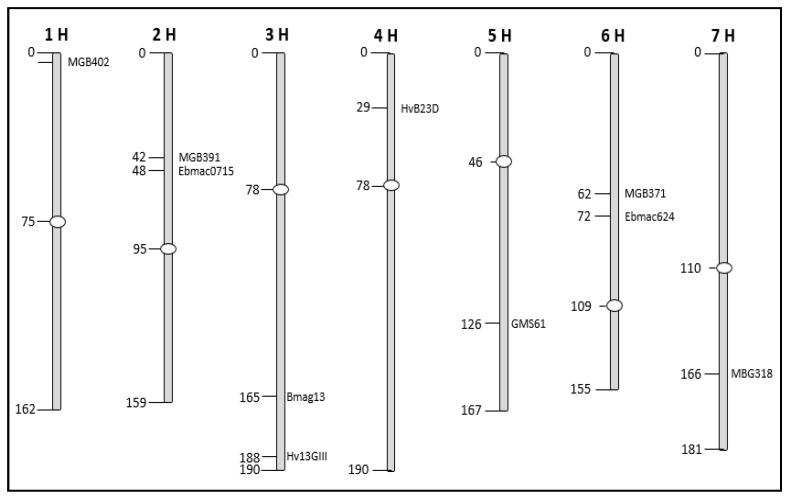
Position of the SSR markers used in the current study on the different chromosomes of the barley genome.

To analyse the phylogenetic relation between all the varieties, the binary matrices of both, RAPD and SSR markers were combined and transformed into a distance matrix using the GenAlex6.5 Excel extension [32]. This matrix was then imported into the Mega software version X [33] to infer phylogenetic tress using the UPGMA or Neighbour Joining as distance-based methods [34]. To obtain insight into the relationship between geographic distribution and genetic distance for the different chromosomes, we calculated distance matrices for the SSR markers located on individual chromosomes, separately (Figure 1). This was conducted for the landraces only.

## 3. Results

### 3.1. Algerian and European Varieties of Barley Cluster Separately

To obtain insight into the phylogenetic relationships between the Algerian varieties with respect to varieties from the Near and Middle East, traditional European landraces, as well as traditional and modern barley cultivars from Germany, we used the same RAPD and SSR markers on a core collection of 30 accession in addition to one accession of the wild ancestor *H. spontaneum* from Armenia. For both, the RAPD and the SSR markers, we obtained well reproducible patterns. In case of RAPD markers, we observed a total of 57 bands, 51 of which were polymorphic (Table 2), with 3 to 8 bands per marker. Polymorphic Information Content (PIC) values were between 0.23 and 0.40, with the highest value scored by the marker OPD02. For SSR analysis (Table 3), the total number of bands was 30, whereby almost all 27 were polymorphic with 2 to 5 bands per marker. The most informative marker was MGB318, followed by MGB 402 and MGB371 with PIC values of 0.72, 0.69 and 0.68, respectively.

**Figure 2:**
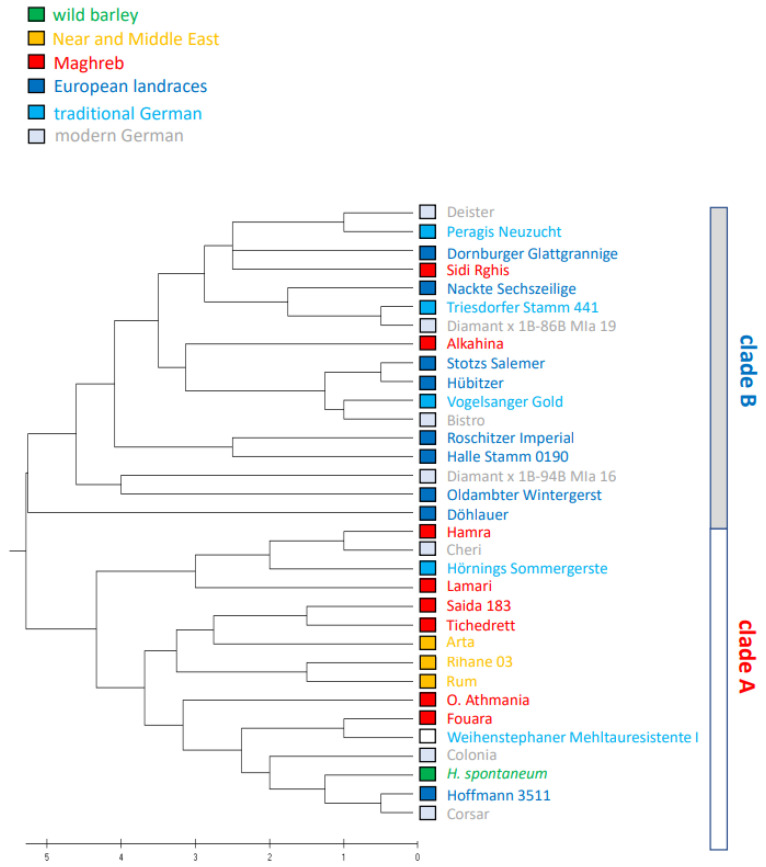
Phylogenetic relationship of Algerian barley varieties in relation to varieties from the Near and Middle East, European landraces, and German cultivars inferred with the UPGMA algorithm based on ten SSR markers, representing all chromosomes.

The SSR markers had been selected such that all chromosomes were symmetrically represented [35], such that the inferred phylogenetic tree would reflect phylogeny appropriately. The resulting tree (Figure 2) displayed two clearly separated clades. Clade A harboured all but two of the Algerian varieties, along with all the traditional varieties from the Middle East, and *H. spontaneum*. Clade B contained exclusively European varieties except the two Algerian varieties Sidi Rghis and Alkahina that derive from crosses with the German variety Express (Table 1) and, thus, is not native to Algeria. Within clade A, the two traditional Algerian landraces Saïda183 and Tichedrett form a twin clade, which is congruent with their origin and autochthonous nature. There are a couple of European varieties that are found in cluster A as well, due to commonalities in ancestry.

For instance, Hamra forms a sister clade with the modern European cultivar Cheri. This is probably due to common ancestors in the complex pedigree of the two varieties: Barberousse derives from Hatif de Grignon, a landrace from Morocco, but also from the French varieties Ager and Ares that both derive from a cross with the traditional varieties Binder (selected from Hanna, a traditional landrace from Bohemia, Czech Republic) and Gull (a traditional landrace from Gotland, Sweden). Cheri derives from the traditional varieties Diamant (Bohemia, Czech Republic), Medusa (deriving from Binder and Gull) and Trumpf (deriving from Hanna). So, their progeny is overlapping, which can explain their close relationship and also their proximity with the traditional German variety Hörnings Sommergerste, deriving from Unstrut/Saale, only 100 km distance from the origin of the Bohemian landrace Hanna. Moreover, the grouping of the German varieties Colonia and Corsar, and the traditional summer barley Hoffmann 3511 with *H. spontaneum* may be linked with a *H. vulgare nigrum* in their ancestry. The ancient landraces from the Near East cluster next to Saïda183 and Tichedrett, especially Arta, a Syrian variety deriving from Arabi Abiad. The varieties Rum (from Jordan) and Rihane 03 (deriving from Athenais, a Greek variety of unknown ancestry, registered in 1939) form a neighbouring pair.

In summary, the SSR-based phylogeny clearly delineates the traditional Algerian varieties from the European varieties and uncover a close link with varieties from the Near East. The few examples that break this pattern can all be explained from the pedigree, where germplasm from different geographic origin was employed.

**Figure 3:**
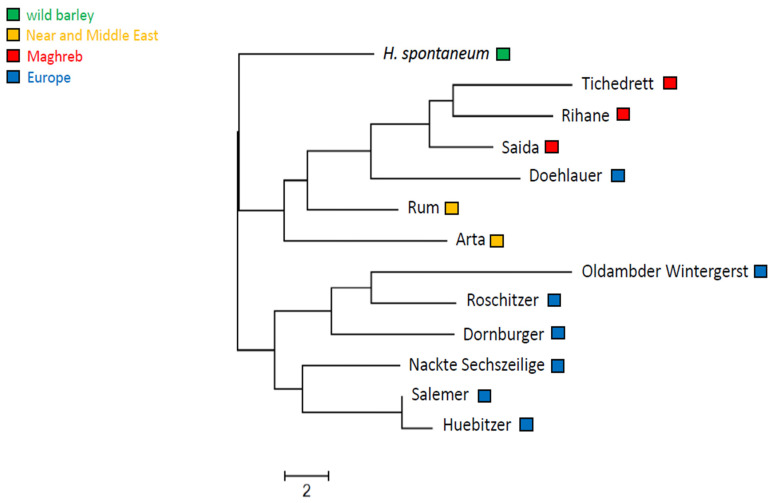
Phylogenetic relationship of Algerian barley varieties in relation to landraces from the Near and Middle East and Europe, as well as wild *H. spontaneum*, inferred with the UPGMA algorithm based on ten SSR markers, representing all chromosomes.

In the next step, we integrated RAPD markers for the entire set of accessions into the SSR-based phylogeny (Supplementary Figure S1). As already seen during a previous study focusing on the analysis of the Algerian varieties alone [35], the RAPD markers reflected geographic proximity rather than pedigree. Therefore, the combination of both markers, while somewhat diluting pedigree relationship, added complementary information. The dilution of pedigree relationship is seen, for instance, for the two Algerian landraces Tichedrett and Saïda183. These were clear twins in the SSR-based tree, but shifted now a bit more apart, while still being close. On the other hand, the higher number of markers also resolved additional clades that appear meaningful. For instance, the wild accession (*H. spontaneum*), originating from Armenia, along with the two accessions Arta and Rum, originating from Syria and Jordan, respectively, clustered now in a clearly defined third clade C. Likewise, the North African varieties Sidi Rghis and Alkahina shifted now from clade B (Figure 3) into the clade A, where the other Algerian varieties are located (Supplementary Figure S1). On the other hand, Lamari moved into the opposite direction.

These differences indicate that in genotypes with a complex pedigree involving North African as well as European progenitors (which holds true for all three mentioned varieties, Sidi Rghis, Lamari and Alkahina) will move, when chromosomes are represented asymmetrically (which only becomes transparent for the SSR markers), depending on the geographic origin of the chromosomes that are overrepresented with respect to the RAPD markers.

These considerations led to the question, whether the inferred complex relationships might become more transparent, if biogeography and phylogenetic relationships of the SSR markers would be considered separately for the individual chromosomes.

### 3.2. Geographic Clustering of Barley Landraces Is Chromosome Dependent

As to obtain insight into the biogeographic relationship of the two Algerian landraces, Saïda183 and Tichedrett, with respect to genotypes from Europe and the Near and Middle East, we omitted the bred varieties with their mostly intricate pedigrees that are usually combining genetic resources from different geographic origin. Instead, we confined the analysis to landraces because these had arisen in a given geographical region, making use of the SSR markers only, since these represented the different chromosomes more or less symmetrically.

In fact, we found a phylogenetic pattern that reflected the geographic distribution of these landraces quite well (Figure 3). As to be expected, the wild *H. spontaneum* was an outgroup to the remaining accessions. The three Maghrebi genotypes formed one clade, the two Near East landraces Rum and Arta were basal to this clade and the majority of the European genotypes were forming a well separated clade with two subclades. These two clades had a partial, but not complete geographic differentiation. The French Salemer, the Middle German Hübitzer and the Southwest German Nackte Sechszeilige comprised one clade, while Roschitzer from Northeast Germany, and Dornburger from Saxonia fell into a second clade, which, however, also contained the Dutch Oldambter. The only genotype that fell out of the pattern, was the landrace Doehlauer from East Prussia (nowadays Poland). This landrace was located between the Maghrebi and the Near East genotypes.

**Figure 4:**
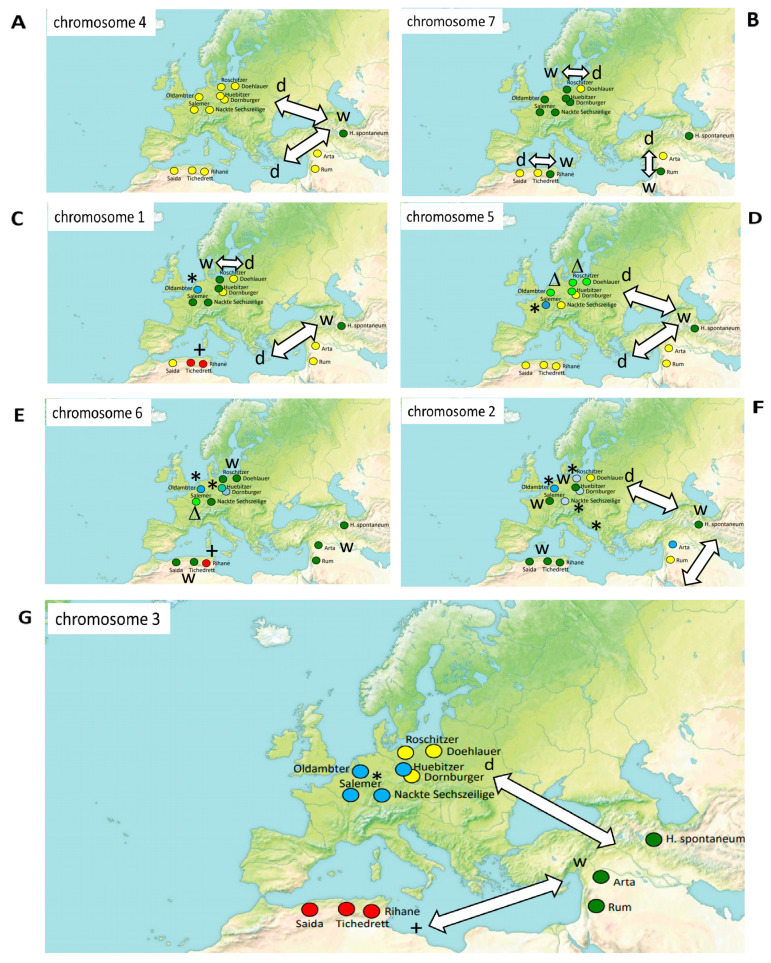
Biogeography of chromosomal diversity as assessed by SSR markers of the respective chromosome, using markers *HvB23D* on chromosome 4 (**A**), *MBG318* on chromosome 7 (**B**), *MGB402* on chromosome 1 (**C**), *GMS61* on chromosome 5 (**D**), *MGB371* and *Ebmac624* on chromosome 6 (**E**), *MGB391* and *Ebmac0715* on chromosome 2 (**F**), *Bmag19* and *Hv13GIII* on chromosome 3 (**G**). w indicates the wild allele found in *H. spontaneum*, d domesticated allele deriving thereof, +, *, Δ indicate alleles secondarily deriving from this domesticated allele.

On the background of a generally close match between genetics and geography, these individual deviations are significant and called for a closer investigation. Since introgression events during the migration and domestication of barley are expected to be reflected as chromosomal recombination, we analysed the phylogenetic relationship for each chromosome individually, making use of polymorphisms of the respective SSR marker located on this chromosome. The resulting clades were mapped on the geographic origin of the landraces to detect potential differences between different chromosomes that would report migration or differentiation events. In fact, the patterns obtained by this approach were strongly dependent on the chromosome under consideration (Figure 4). In fact, the resulting patterns were highly dependent on the respective chromosome:

For instance, marker *HvB23D*, located on chromosome 4 differentiated all landraces from *H. spontaneum* (Figure 4A, Supplementary Figure S2A) while marker *MBG318* on chromosome 7 (Figure 4B, Supplementary Figure S2B) differentiated a majority (including *H. spontaneum*) from a minority comprising the two Algerian landraces Saïda183 and Tichedrett, the Near East landrace Arta, and the East Prussian landrace Doehlauer. Interestingly, Rihane, which is a cross of a landrace from Morocco with European varieties, differs from Saïda183 and Tichedrett and belongs to the bigger group that shares the allele with *H. spontaneum*.

Marker *MGB402*, located on chromosome 1, produces a more complex pattern (Figure 4C, Supplementary Figure S2C). Here, the European accessions have retained the wild allele from *H. spontaneum*, except again the East Prussian landrace Doehlauer, and the East German landrace Dornburger that share an allele with the Near East landraces Arta and Rum, but also with Saïda183. In contrast, Tichedrett shares a specific allele with Rihane that is not found outside Algeria. A further private allele is the historical Dutch landrace Oldambter.

Marker *GMS61* located on chromosome 5, differentiates *H. spontaneum* from all domesticated varieties (Figure 4D, Supplementary Figure S2D). While this matches the situation for chromosome 4, chromosome 5 further diverges within the domesticated varieties. The three varieties from the Maghreb, and the two varieties from the Near East share their allele with the East German landrace Dornburger and the South German Nackte Sechszeilige. The North German landraces as well as the Dutch Oldambter harbour a different allele, and the landrace Salemer, originating from France, has its private allele.

Markers *MGB371* and *Ebmac624*, both on chromosome 6 (Figure 4E, Supplementary Figure S2E), produced a pattern, where the original *H. spontaneum* allele was preserved throughout the Near East and the Maghreb (with exception of Rihane that showed a private allele), while in European landraces, there had been considerable differentiation. The original allele was still found in Doehlauer, Roschitzer, and Nackte Sechszeilige, while the other landraces all harboured their own, private alleles.

A variation in this pattern was seen for chromosome 2, reported by the markers *MGB391* and *Ebmac0715* (Figure 4F, Supplementary Figure S2F). Here, the *H. spontaneum* allele was found in all three landraces from the Maghreb (including Rihane), as well as in the French landrace Salemer and the German landrace Hübitzer. The Near East landrace Rum and the East Prussian Doehlauer shared a different allele, and the Near East landrace Arta shared a further allele with the Dutch landrace Oldambter. The other three German landraces (Roschitzer, Dornburger and Nackte Sechszeilige) harboured a fourth allele.

The clearest geographic separation was observed for chromosome 3 (monitored by the markers *Bmag19* and *Hv13GIII*). Here, the Near East landraces Arta and Rum shared the ancestral allele from *H. spontaneum*, while all three Maghrebi accessions formed a separate clade (Figure 4G, Supplementary Figure S2G). Among the European landraces, a Western group including the Dutch Oldambter, the French Salemer, and the Southwest German Nackte Sechszeilige, and the Middle German Hübitzer could be discerned from an Eastern group with Roschitzer from Northeast Germany, Doehlauer from East Prussia and Dornburger from Saxonia.

## 4. Discussion

In the current work, we investigated the phylogenetic relationship of two barley landraces from Algeria, Saïda183 and Tichedrett with respect to a panel of barley varieties from Europe and the Near East as well as a *H. spontaneum* accession from Armenia using RAPD and SSR markers. We show that, on the base of SSR-based phylogeny, the Algerian varieties cluster clearly with those from the Near East but separate from the European varieties. The pattern inferred from RAPD markers, partially confirms these findings, albeit with a higher degree of ambiguity. We use then a subset of the panel, comprising only traditional landraces that can be linked to a specific geographic region to construct the phylogenetic relationship for each chromosome individually, making use of the fact that the set of SSR markers used in the current study, was more or less symmetrically distributed over the seven chromosomes of barley. This approach uncovers qualitative differences in the biogeographical patterns with a gradient of resolution. One endpoint is represented by marker *HvB23D* (chromosome 4), separating the wild *H. spontaneum* from all domesticated genotypes, while on the other end of the cline, markers *Bmag19* and *Hv13GIII* (chromosome 3) identify four distinct geographic clusters. The remaining markers can be placed on different positions in-between with respect to their geographic resolution.

These findings lead to the following questions that will be discussed below: To what extent do SSR and RAPD markers tell the same story and to what extent do they address different facets? What can we learn when we consider different chromosomes separately? What can we learn about the origin of the Algerian landraces and their relationship with traditional barley varieties from Europe and the Near East?

### 4.1. Symmetry Versus Asymmetry—Why SSR and RAPD Address Different Facets

Fingerprinting strategies, such as Random Amplified Polymorphic DNA (RAPD) or Small Sequence Repeats (SSR) are used as cost-effective strategies to discriminate varieties of a given crop, or to infer their phylogenetic relationships. Since RAPD uses standardised sets of arbitrary primer pairs, it can even be used in cases where there is little genetic knowledge [36]. The drawbacks are the low annealing temperatures rendering this method vulnerable to experimental noise. In contrast to RAPD, fingerprinting by SSR allows the selection of targets, whose location in the genome is known [37,38]. In our case, we selected these markers such that all chromosomes were represented reducing sampling bias, which in case of RAPD markers can only be avoided using a large number of markers. The fact that the phylogeny (Supplementary Figure S2), and the geographical patterns of the SSR alleles (Figure 4) were strongly dependent on the chromosome, where the respective marker was located demonstrates that the *caveat* about sampling bias was valid.

These considerations lead to the question, whether RAPD are just a poor and preliminary alternative, and should be abandoned altogether as soon as SSR markers and information on their chromosomal location become available. The answer to this question is negative—RAPDs can give information by their own right. In our previous study, addressing different barley landraces from North Africa [35], we found that, while RAPD provided only a poor representation of pedigree, they reflected geographic origin of these landraces at better resolution as compared to SSR markers, a phenomenon observed also in a couple of other studies [39,40]. Since landraces are usually linked with a particular region with a limited gene flow to neighbouring regions (for instance, by trading of seeds), and since RAPDs are selected for maximal polymorphism among the analysed samples, the markers of adjacent regions will coincide more strongly as compared to SSR markers that had been selected from symmetric representation of all chromosomes. The sampling bias of RAPDs can, thus, zoom into otherwise overlooked differences deriving from geography.

On this background it is worth to consider the difference between the phylogenies inferred by the SSR markers alone (Figure 2) and the combined use of SSR and RAPD markers (Supplementary Figure S1).

The two clades emerging from the SSR markers (Figure 2) seem to reflect the pedigree in the first place, including even minor details of relationship phylogeny. The two autochthonous Algerian landraces Saïda183 and Tichedrett cluster with traditional varieties from the Middle East, as well as with *H. spontaneum*, and all but two Algerian varieties, while the majority of European varieties cluster in a well separated clade. Even the apparent exceptions to the rule, the Algerian varieties Sidi Rghis and Alkahina that are located in the European clade, are in fact derivatives of crosses with the German variety Express and, thus, are, in fact, of European origin. Likewise, the few European varieties that are interspersed in cluster A, do this due to overlapping pedigree, because germplasm from the Near East and Ethiopia have been used in breeding programmes targeted to resistance against Powdery Mildew.

The addition of RAPD markers (Supplementary Figure S1), while diluting the clear phylogenetic patterns seen for the SSR markers alone, added interesting information of biogeographic nature. A third clade, comprising *H. spontaneum* from Armenia, the Syrian landrace Arta, and the Jordanian landrace Rum became manifest. Moreover, the North African cluster could now be differentiated reflecting geographic patterns.

In the summary, our findings demonstrate that SSR markers are superior to RAPDs in reflecting phylogenetic relationships. The reason is not the differences in quality (both approaches use size polymorphisms), but the possibility to select SSR markers with respect to symmetric representation of chromosomes. In case of RAPDs, where the chromosome location is not known, asymmetric representation is very likely, if one does not use a very high number of markers. However, this asymmetry can help to coarse-grain geographic patterns, as reflected by the differentiation between Middle East and Maghreb landraces detected, when RAPD and SSR markers are merged (Supplementary Figure S1). In other words, sampling bias is not only a factor to be considered, but it can, by itself, be used to learn something about the history of barley domestication, as will be discussed in the following.

Regarding the RAPD experimental limitations, achieving consistent results requires rigorous standardisation, careful DNA purification, optimised PCR conditions, and well-defined electrophoresis settings [35]. Addressing these challenges would also necessitate using significantly more primer pairs than are typically employed.

### 4.2. Make Bias Useful—What to Learn from Looking at Chromosomes Independently

**Figure 5:**
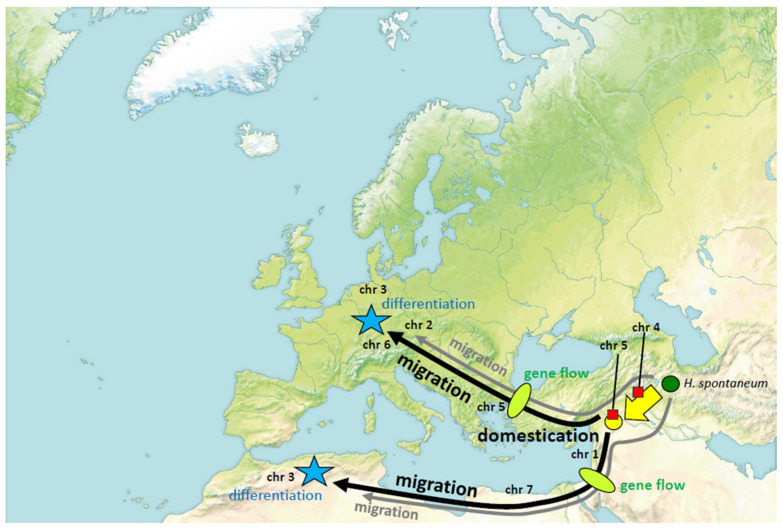
Processual model on phylogenetic relationships between barley landraces from the Middle East, Maghreb, and Central Europe based on asymmetric relations between individual chromosomes. Inferred temporal sequence of major geographic differentiation in individual chromosomes is indicated by the abbreviation. Red squares indicate differentiation linked to domestication, green ellipses indicate gene flow between domesticated barley and contaminating *H. spontaneum*, blue stars represent geographical differentiations occurring late, upon arrival in the target region.

The selection of SSR markers that are representing all chromosomes allows to infer phylogenetic relationships. On the other hand, RAPD markers are prone to sampling bias, which can help to coarse-grain geographic relationships, and this may help to see additional facets, although these patterns are difficult to interpret, since their location on the chromosome is not known. A third strategy would be to introduce deliberate asymmetry, by analysing the SSR markers separately for the individual chromosomes, and to project this upon the geographic distribution of barley landraces. In fact, when we pursued this strategy, we found quite distinct patterns, depending on the respective chromosome, with a gradient ranging from marker *HvB23D* on chromosome 4 reflecting the difference between domestication and wild *H. spontaneum* till markers *Bmag19* and *Hv13GIII* on chromosome 3 revealing clearly separated geographic clusters (Near and Middle East, Western Europe, Eastern Europe, Maghreb) and delineating the Maghreb landraces from the rest. The geographic patterns emerging from this analysis can be interpreted in frame of a working model, where introgression of individual chromosomes from wild into domesticated barley occurred concomitantly with migration of domestic barley along with wild barley (as contaminant of seed stocks) due to human migration and cultural exchange (Figure 5):

For chromosome 4, all domesticated varieties share the same allele diverging from *H. spontaneum*, indicating that this split occurred during domestication. Chromosome 5 diversified only shortly after, still before the migration to the Maghreb and Europe, since the same allele (differing from *H. spontaneum*) is shared in barley from the Near East, Maghreb, and Southern Europe. Two additional alleles, in France and Northern Germany, seemed to have arisen later, after arrival. Chromosome 1 is the first, where private alleles of Maghrebi barley occur, it seemed to have diversified after barley has already started its migration to Europe and the Maghreb. Chromosomes 2 and 6 have apparently differentiated on the journey to Europe, because, here, the Maghrebi barley still has retained the wild allele found in *H. spontaneum*. For chromosome 7, instead, the Maghrebi accessions seem to have diverged, showing a link to the accession Rum from Jordan, while the European accessions seem to have retained the original wild allele. The clearest geographical differentiation is seen for chromosome 3. It is, thus, straightforward to conclude that chromosome 3 differentiated late, after arrival in the respective region.

These findings contribute to an ongoing larger debate on crop domestication. As pointed out in a conceptual editorial by Robin Allaby [41] the combination of Darwin’s genetic viewpoint on domestication of plants and animals along with Vavilov’s finding that crop plants can often be traced back to defined centres of origin, has led to the notion that domestication of a crop must necessarily be seen as a single historic event. Barley seems to challenge this concept as demonstrated by an extensive genetic study across several hundreds of barley landraces using a large set of single-nucleotide polymorphisms and Bayesian clustering [42]. This study reveals four geographically separated clusters—one extending from the Fertile Crescent to Central Europe, one Mediterranean comprising the Maghreb landraces, a third, well separated cluster centering to Ethiopia, and a fourth Himalayan cluster extending from Iran over Pakistan, North India to India and East Asia. While this central finding is consistent with the alternative view that barley was independently domesticated several times in different regions, there are some details that add ambiguity to this seemingly clear-cut image. For instance, the Mediterranean cluster has invaded the Fertile Cresent/Central European cluster and split it into halves. Furthermore, barley landraces from Mongolia, Korea, and Kyushu show a strong overlap with this Mediterranean cluster, while the Himalayan cluster is also found at the west coast of Norway. These exceptions to the rule highlight an important factor that is often overlooked: human migration.

A system of seed quality and purity control, standard in modern seed production, did not exist during the Neolithic Revolution. Therefore, migration of the new technology of barley agriculture was most likely meaning that the seeds of domesticated barley were mixed with wild *H. spontaneum* as seed contaminant. Introgression from local populations of wild barley with its wide geographic distribution [43] contributed further to the differentiation of geographically separated clusters. A similar situation has been proposed for the spread of domesticated grapevine after dual domestication in the Caucasus and the Near East along with the postglacial spread of its wild ancestor, the European Wild Grapevine, from its Pleistocene refugium in the Caucasus region [44].

The wild barley ancestor, *H. spontaneum*, shows a low rate of outcrossing of around 2% [45], which might contribute to the pronounced regionality found for the markers on chromosome 3. For markers on chromosome 4, where all landraces share the same allele differentiated from wild barley, the large geographical distribution might be explained by shared mutational events at the time of domestication. Interesting are cases, where the pattern of geographical distribution does not match the genetic patterns, for instance for the chromosomes 5, 6, and 2 (Figure 4D–F). Here, Maghrebi landraces match with subsets of Central European landraces while adjacent European landraces harbour different alleles. Such patterns can neither be explained by a shared event during domestication, nor by local differentiation sustained by inbreeding, but most reflect events that had happened during the migration of barley. Some of these alleles are also shared with *H. spontaneum*, but not with the domesticated Near East landraces. The most straightforward explanation is that of introgression during migration. The low outcrossing rate of barley renders introgression a rare, but by no means an impossible event. It will, thus, depend on the distance of seed dispersal, to what extent introgression into domesticated barley can take place. A study, where the geographical distribution of alleles for nine nuclear loci was investigated in an extensive phylogenetic study allowed for estimating parameters of seed dispersal in wild barley, reaching the astonishing value of more than 3000 km [46], which lends further support to a processual model of migration and introgression.

Our study builds on these foundational insights, exploring genetic diversity within Algerian barley landraces, which have been relatively underexplored. The focusing specifically on landraces, aimed to provide more precise and defined insights into their importance. In summary, these results not only reinforce a multi-regional model of barley domestication but also highlight the enduring role of wild barley in shaping the diversity of modern landraces. This work advances the understanding of crop domestication by integrating genetic evidence, historical human migration patterns, and the unique contributions of local wild populations. It highlights the critical role of landraces as reservoirs of genetic diversity and adaptive traits, providing valuable resources for future breeding programmes and agricultural innovation.

## 5. Conclusions and Outlook

Utilising a combination of symmetrically distributed Simple Sequence Repeats (SSR) and geographically coarse-graining Random Amplified Polymorphic DNA (RAPD) markers, we investigate the phylogenetic relationship of the Algerian landraces Saïda183 and Tichedrett with respect to landraces and modern varieties from Europe, the Near East, and wild barley from Armenia. By investigating geographic patterns separately for markers on individual chromosomes, we find different patterns ranging from domestication-related polymorphisms till strict regional differentiation. Notably, for some chromosomes, the alleles shared with the wild ancestor but not with other domesticated accessions can be found across geographic clusters indicating introgression during different migration phases. These shared alleles, likely subject to selective pressures, underscore the adaptive potential embedded in the genetic diversity of Algerian landraces.

To deepen understanding, future studies should strengthen the use of markers and incorporate additional ones, such as single-nucleotide polymorphisms (SNPs), covering chromosomes with contrasting geographic patterns (e.g., chromosomes 3 and 4) and also include more landraces from the intermediate regions, especially Turkey, the Balkan region, as well as Libya and Egypt. Given the role of human migration’s impact on barley diversity, a real understanding will require an interdisciplinary approach that integrates molecular genetics with archaeology, cultural anthropology, and traditional barley cultivation practises.

This approach is essential for leveraging the adaptive genetic diversity in these landraces for molecular breeding, advancing the development of barley varieties resilient to climate and environmental stresses.

## Figures and Tables

**Table 1:** Barley accessions used in the current study. Origins are indicated by the international country abbreviations.

Name	Taxon	Origin	Source	Pedigree	Reference
**Wild barley**					
*H. spontaneum*	*H. spontaneum K.Koch*	AM	collected	Erebuni Nature Reservate (district Kotayk marz)	[14]
**Autochthonous landraces of the Near and Middle East**					
Rum	*H. vulgare* var. *hexastichon*	JO	?	probably deriving from Arabi Aswad or Arabi Abiad	[15]
Arta	*H. vulgare* var. *distichon*	SY	ICARDA	deriving from Arabi Abiad	[16]
**Autochthonous landraces from Algeria**					
Saïda183	*H. vulgare* var. *hexastichon*	DZ	ITGC	local landrace, released in 1995, winter barley, around 90% of Algerian barley area	[17]
Tichedrett	*H. vulgare* var. *hexastichon*	DZ	ITGC	local landrace, released in 1997, winter barley, around 10% of Algerian barley area	[17]
**Other Algerian genotypes**					
Sidi Rghis	*H. vulgare* var. *hexastichon*	DZ	ITGC	Express xAlonda01	[18]
Alkahina	*H. vulgare* var. *hexastichon*	DZ	ITGC	Nadawa × Rihane 03 × Express	[18]
Hamra	*H. vulgare* var. *hexastichon*	DZ	ITGC	Barberousse [(Hauter × (Hatif de Grignon × Ares)) × Ager]	[19,20]
Fouara	*H. vulgare* var. *hexastichon*	DZ	ITGC	Sétif	[18]
O.Athmania	*H. vulgare* var. *hexastichon*	DZ	ITGC	Saida/CitaS/Apm/copal/Bon/5/Rihane3	[18]
**European Landraces**					
Döhlauer	*Hordeum vulgare* L. *convar. vulgare* var. *hybernum Viborg*	DE	IPK	landrace from East Prussia, summer barley	[21]
Dornburger Glattgrannige	*Hordeum vulgare* L. *convar. distichon* (L.) *Alef.* var. *medicum Körn.*	DE	IPK	landrace from Germany (Saale), summer barley	[21]
Halle Stamm 0190	*Hordeum vulgare* L. *convar. vulgare* var. *hybernum Viborg*	DE	IPK	winter barley, source Technical University of Munich	[21]
Hoffmann 3511	*Hordeum vulgare* L. *convar. distichon* (L.) *Alef.* var. *nutans (Rode) Alef.*	DE	IPK	landrace from Germany. Summer barley	[21]
Oldambter Wintergerst (KIT 9061)	*H. vulgare* var. *zeocriton*	NL	BG Klagen-furt	landrace from Frisia. De Heimanshof, Hoofdorp, Netherlands Frisian barley	[22]
Hübitzer	*Hordeum vulgare* L. *convar. vulgare* var. *hybernum Viborg*	DE	IPK	selected in 1916 in Hübitz, South Harz. Released in 1948 by Hansen	[21]
Nackte Sechszeilige	*Hordeum vulgare* L. *convar. vulgare* var. *subnudipyramidatum (Orlov) Mansf.*	DE	IPK	summer barley, in use before 1945, source University of Hohenheim	[21]
Roschitzer Imperial	*Hordeum vulgare* L. *convar. distichon* (L.) *Alef.* var. *erectum (Rode) Alef.*	DE	IPK	Niederlausitzer Saatzucht before 1945, summer barley, grown in the Uckermark, now used for vintage Whisky, source University of Hohenheim	[21]
Stotzs Salemer	*Hordeum vulgare* L. *convar. vulgare* var. *hybernum Viborg*	DE	IPK	selected from a dense-eared French landrace. release in 1916 by Stotz	[20]
**Traditional German genotypes**					
Triesdorfer Stamm 411	*Hordeum vulgare* L. *convar. distichon* (L.) *Alef.* var. *nutans (Rode) Alef.*	DE	IPK	winter barley, was used in the Agricultural Research Station Triesdorf as source for varieties released in the 1950s.	[21]
Hörnings Sommergerste	*Hordeum vulgare* L. *convar. distichon* (L.) *Alef.* var. *nutans (Rode) Alef.*	DE	IPK	Gebrüder Hörning, Roßleben (Unstrut)	[23]
Peragis Neuzucht	*Hordeum vulgare* L. *convar. distichon* (L.) *Alef.* var. *nutans (Rode) Alef.*	DE	IPK	Heils Franken (landrace from Frankonia) × Bavaria (landrace from Niederbayern)	[20]
Vogelsanger Gold	*Hordeum vulgare* L. *convar. vulgare* var. *hybernum Viborg*	DE	IPK	(Isaria × H204(H. spontaneum.nigrum)) × WG 5	[21]
Weihenstephaner Mehltauresistente I	*Hordeum vulgare* L. *convar. distichon* (L.) *Alef.* var. *nutans (Rode) Alef.*	DE	IPK	Criewener 503 × Pflug’s Intensiv	[20]
**Modern German genotypes**					
Bistro	*Hordeum vulgare* L. *convar. distichon* (L.) *Alef.* var. *nutans (Rode) Alef.*	DE	IPK	(Colambo × Astrid) × Angora	[21]
Cheri	*Hordeum vulgare* L. *convar. distichon* (L.) *Alef.* var. *nutans (Rode) Alef.*	DE	IPK	Trumpf × (Medusa × Diamant)	[21]
Colonia	*Hordeum vulgare* L. *convar. vulgare* var. *hybernum Viborg*	DE	IPK	Kiruna × Trumpf	[20]
Corsar	*Hordeum vulgare* L. *convar. vulgare* var. *hybernum Viborg*	DE	IPK	Ally × Asorbia	[20]
Deister	*Hordeum vulgare* L. *convar. distichon* (L.) *Alef.* var. *nutans (Rode) Alef.*	DE	IPK	Abed 89 × Minerva	[20]
Diamant × 1B-94B MIa 16	*Hordeum vulgare* L. *convar. distichon* (L.) *Alef.* var. *nutans (Rode) Alef.*	DE	IPK	Summer barley, 1988. Czech Republic. released in 1965 by Branisovice	[21]
Diamant × 1B-86B MIa 19	*Hordeum vulgare* L. *convar. distichon* (L.) *Alef.* var. *nutans (Rode) Alef.*	DE	IPK	Summer barley, 1988. Czech Republic. mutant from Valtický	[21]

**Table 2:** Oligonucleotide primers used for the Randomly Amplified Polymorphic DNA (RAPD) analysis, total number of bands (TB) generated by these markers and the number of bands, where polymorphism was detected (PB) along with the calculated polymorphism information content (PIC).

Marker	Primer	TB	PB	PIC	Reference
AF14	5′-GGTGCGCACT-3′	5	5	0.29	[24]
By15	5′-CTCACCGTCC3′	6	5	0.38	[25]
LG13	5′-GTTGCCAGCC-3′	5	4	0.33	[24]
OPAM02	5′-ACTTGACGGG-3′	7	6	0.23	[26]
OPC07	5′-GTCCCGACG-3′	3	2	0.24	[26]
OPC13	5′-AAGCCTCGCT-3′	7	7	0.25	[26]
OPD02	5′-GGACCCAACC-3′	5	5	0.40	[24]
PKAT17	5′-AGGGACTGCT-3′	5	5	0.33	[26]
UBC402	5′-CCCGCCGTTG-3′	8	6	0.26	[25]
UBC534	5′-CACCCCCTGC-3′	6	6	0.37	[25]

**Table 3:** Oligonucleotide primers used for the Simple Sequence Repeat (SSR) analysis along with their chromosome locations (Chr); total number of bands (TB) generated by these markers and the number of bands, where polymorphism was detected (PB) along with the calculated polymorphism information content (PIC).

Marker		Sequence	Chr	TB	PB	PIC	Reference
Bmag13	Fw Rev	5′-AAGGGGAATCAAAATGGGAG-3′ 5′-TCGAATAGGTCTCCGAAGAAA-3′	3H	2	3	0.62	[27]
Ebmac0715	Fw Rev	5′-GCGAACATTGTCATGTTAGTA-3′ 5′-TGTCATGCCAGACCTATG-3′	2H	2	1	0.44	[28]
EBmac624	Fw Rev	5′-AAAAGCATTCAACTTCATAAGA-3′ 5′-CAACGCCATCACGTAATA-3′	6H	2	2	0.49	[27]
GMS1	Fw Rev	5′-CTGACCCTTTGCTTAACATGC-3′ 5′-TCAGCGTGACAAACAATAAAGG-3′	7H	2	2	0.42	[28]
HV13GEIII	Fw Rev	5′-AGGAACCCTACGCCTTACGAG-3′ 5′-AGGACCGAGAGTGGTGGTGG-3′	3H	2	2	0.49	[27]
HVB23D	Fw Rev	5′-GGTAGCAGACCGATGGATGT-3′ 5′-ACTCTGACACGCACGAACAC-3′	4H	4	3	0.26	[28]
MGB318	Fw Rev	5′-CGGCTCAAGGTCTCTTCTTC-3′ 5′-TATCTCAGATGCCCCTTTCC-3′	7H	4	3	0.72	[27]
MGB371	Fw Rev	5′-CACCAAGTTCACCTCGTCCT-3′ 5′-TTATTCAGGCAGCACCATTG-3′	6H	5	5	0.68	[28]
MGB391	Fw Rev	5′-AGCTCCTTTCCTCCCTTCC-3′ 5′-CCAACATCTCCTCCTCCTGA-3′	2H	2	2	0.49	[27]
MGB402	Fw rev	5′-CAAGCAAGCAAGCAGAGAGA-3′ 5′-AACTTGTGGCTCTGCGACTC-3′	1H	5	5	0.69	[28]

## Data Availability

Data is contained within the article and Supplementary Materials.

## Supplementary Materials

The following supporting information can be downloaded at: https://www.mdpi.com/article/10.3390/cimb46120852/s1. Figure S1: Phylogenetic relationship of Algerian barley varieties in relation to varieties from the Near and Middle East, European landraces, and German cultivars inferred with the UPGMA algorithm based on ten SSR markers, representing all chromosomes integrating genetic distances from ten RAPD markers. Figure S2: Phylogenetic relationship in dependence of the respective chromosome, using markers *HvB23D* on chromosome 4, *MBG318* on chromosome 7, *MGB402* on chromosome 1, *GMS61* on chromosome 5, *MGB371* and *Ebmac624* on chromosome 6, *MGB391* and *Ebmac0715* on chromosome 2, *Bmag19* and *Hv13GIII* on chromosome 3.
