# Supplementary material for: Genetic Assessment and Positioning of Algerian Barley Landraces with Respect to Landraces from the Middle East and Europe Using RAPD and SSR Markers
Source: Curr Issues Mol Biol. 2024 Dec 17;46(12):14226–43. doi: 10.3390/cimb46120852 (PMC11674917; doi:10.3390/cimb46120852)

Supplementary Figure S1. Phylogenetic relationship of Algerian barley varieties in relation to varieties from the Near and Middle East, European landraces, and German cultivars inferred with the UPGMA algorithm based on ten SSR markers, representing all chromosomes integrating genetic distances from ten RAPD markers.

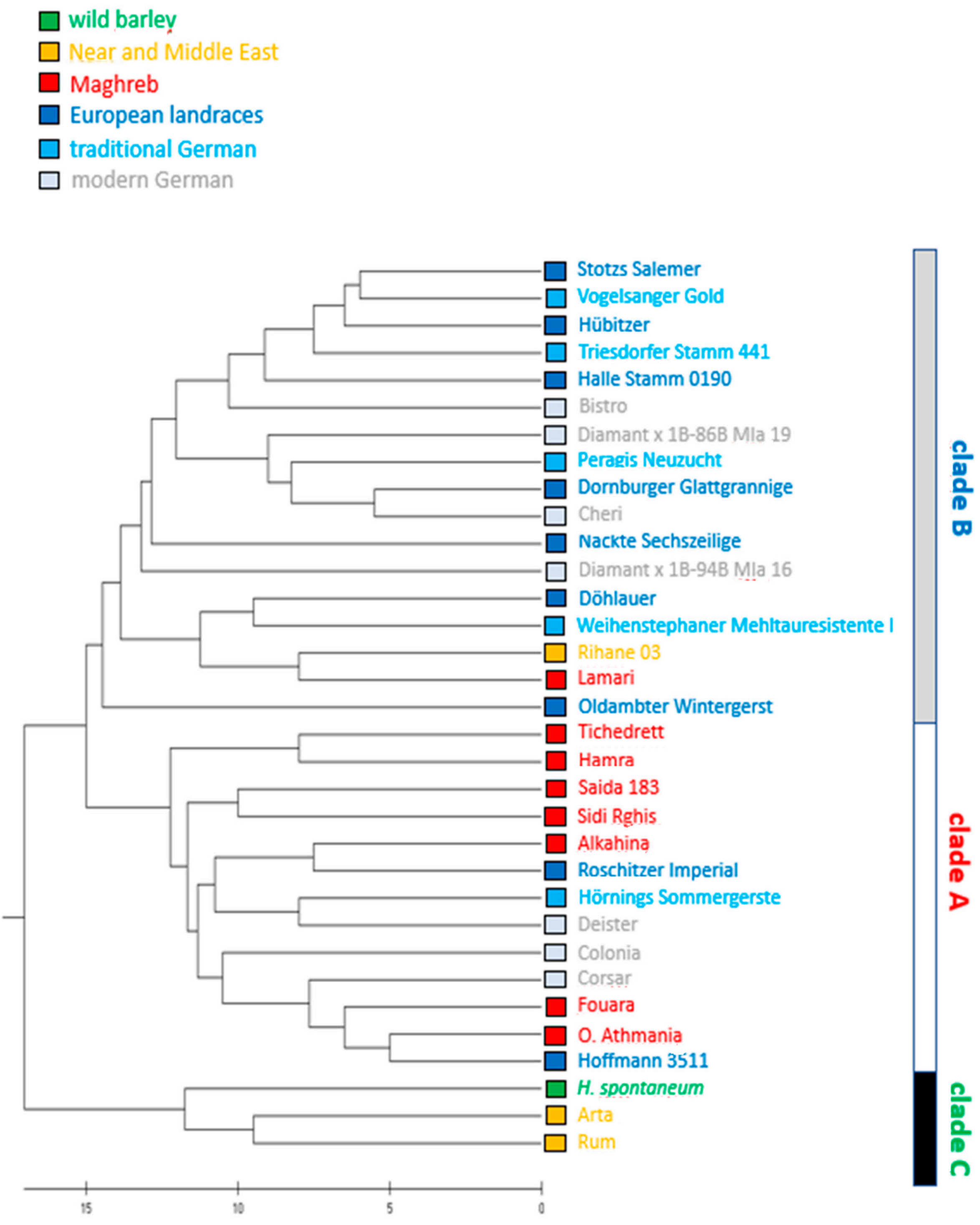

Supplementary Figure S2. Phylogenetic relationship in dependence of the respective chromosome, using markers HvB23D on chromosome 4, MBG318 on chromosome 7, MGB402 on chromosome 1, GMS61 on chromosome 5, MGB371 and Ebmac624 on chromosome 6, MGB391 and Ebmac0715 on chromosome 2, Bmag19 and Hv13GIII on chromosome 3.

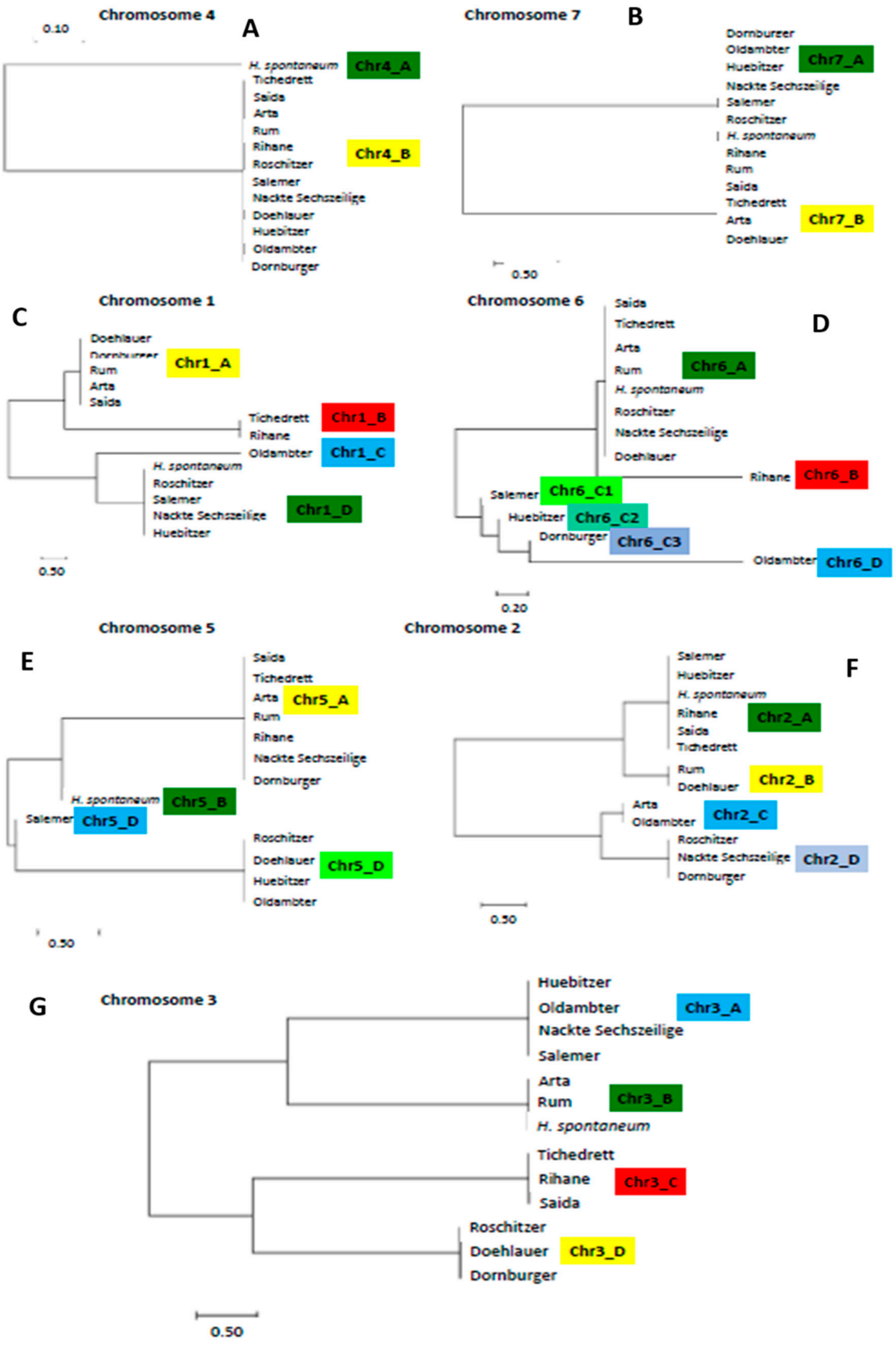

Supplement: Supplementary file 1 [file cimb-46-00852-s001.zip › cimb-3314805-supplementary.pdf]
